# Cervical Lymph Node Metastases in Oral Squamous Cell Carcinoma—How Much Imaging Do We Need?

**DOI:** 10.3390/diagnostics10040199

**Published:** 2020-04-02

**Authors:** Johannes Laimer, Anke Lauinger, Otto Steinmassl, Vincent Offermanns, Astrid E. Grams, Bettina Zelger, Emanuel Bruckmoser

**Affiliations:** 1 University Hospital for Cranio-Maxillofacial and Oral Surgery, A-6020 Innsbruck, Austria; 2 University Hospital for Neuroradiology, A-6020 Innsbruck, Austria; 3 Institute of Pathology, Medical University of Innsbruck, A-6020 Innsbruck, Austria; 4 Private Practice for Oral and Maxillofacial Surgery, A-5020 Salzburg, Austria

**Keywords:** oral squamous cell carcinoma, lymph node metastases, ultrasound, computed tomography, magnetic resonance imaging, positron emission tomography

## Abstract

Cervical lymph node metastases in oral squamous cell carcinoma (OSCC) are key predictors of disease specific survival. It was therefore the aim of this study to evaluate how much imaging is minimally needed for reliable and efficient identification of cervical lymph node metastases. In this retrospective cross-sectional study, results (metastasis yes/no) of ultrasound (US), computed tomography (CT), magnetic resonance imaging (MRI), and positron emission tomography (PET) were compared to the final histopathological results of the corresponding neck dissection (ND) specimens (metastasis yes/no). A score was calculated to account for cervical lymph node size, shape, clustering, peripheral enhancement, hilus sign, architecture, blood flow, and central necrosis. Sensitivity and specificity were analyzed for each imaging technique separately. In 164 patients diagnosed with OSCC, 96 underwent uni- or bilateral ND (122 ND in total). One hundred percent sensitivity was achieved by CT+MRI, MRI+PET, US+CT+MRI, US+MRI+PET, CT+MRI+PET, and US+CT+MRI+PET. The highest specificity was realized by US with 79% (95% CI [0.698–0.890]). Specificity for CT+MRI and PET+MRI was 51% (95% CI [0.359–0.665]) and 70% (95% CI [0.416–0.984]), respectively. Regarding 100% sensitivity with acceptable specificity, the combination of CT+MRI or PET+MRI appeared to be suitable for staging cervical lymph nodes in primary OSCC.

## 1. Introduction

Oral squamous cell carcinoma (OSCC) is the 15th most frequent tumor worldwide in men and women. Unfavorably, the incidence is increasing with approximately 300,000 new reported cases annually, or 2.1% of the world total [1]. Risk factors include smoking, especially in combination with heavy alcohol consumption, nonvegetarian diet, poor oral hygiene, poor dentition [2], and in rare cases infections with human papilloma virus (HPV) [3].

The prognosis of OSCC is generally poor with an overall survival rate of less than 60% [4], as well as an estimated recurrence rate of 30% [5]. In a recent paper [6], the authors pointed out that 50% of OSCC affects the tongue and shows aggressive clinical behavior with a five-year survival rate of only 60%. The persistence of poor survival in this subgroup underscores the need for better prognostic tools, such as the lymph node ratio (LNR) or improved patient stratification.

Cervical lymph node metastases in OSCC are a strong factor in terms of increased recurrence and disease-specific and overall death [7]. Neck metastasis confers up to 50% reduced overall survival along with increased metastatic lymph node burden [8]. Therefore, diagnosis of the presence and extent of lymph node metastases is key for the appropriate management of the disease.

Currently, in clinical routine there are various imaging techniques available for tumor staging, such as ultrasound (US), computed tomography (CT), magnetic resonance imaging (MRI), and positron emission tomography (PET) which can be combined with CT (PET-CT) or even MRI (PET-MRI).

Contrary to the prospective study by Adams et al. [9] who reported higher sensitivity and specificity for PET examinations compared to US, CT, or MRI, such statistically significant differences were neither found in the prospective clinical trials by Stuckensen et al. [10] and Stoeckli et al. [11] nor in the retrospective studies by Pohar et al. [12] and Yoon et al. [13]. Interestingly, the studies by Roh et al. [14] and Nguyen et al. [15] showed superiority of PET-CT over CT or MRI. Especially with regard to occult metastases, PET-CT was shown to be superior to CT or MRI in a prospective study by Roh et al. [14]. Concerning contralateral and bilateral lymph node metastases, superiority of PET-CT over CT and MRI was reported by Nguyen et al. [15] in a retrospective study.

Referring to Adams et al. [9], optimal diagnostics could potentially be achieved by combining both functional (PET) and morphological (US, CT, MRI) imaging modalities. In a retrospective study, Yoon et al. [13] reported higher sensitivity without losing specificity for combinations of US, CT, MRI, and PET-CT compared to single investigations. However, results did not reach statistical significance.

According to Stuckensen et al., Ng et al., and Stoeckli et al. [10,11,16], there is currently no imaging technique available to reliably replace elective neck dissection. However, Yamazaki et al. [17] suggested that the precision of PET exams regarding lymph nodes ≥10 mm and the lower false positive rate compared to CT could potentially avoid unnecessary neck dissections.

The choice of imaging modalities for the diagnostic workup has major implications on health care spending. This is of high importance regarding the fact that in recent years, available funds and resources have become more and more restricted in most countries. Besides, issues such as radiation exposure in CT and PET examinations have to be taken into consideration.

The purpose of this study was to determine the extent of diagnostic imaging needed for valid detection of cervical lymph node metastases in OSCC subjects in a practical clinical setting.

The specific aim of the study was to compare the outcome of four different imaging modalities (US, CT, MRI, PET) with reference to the diagnostic gold standard (i.e., histopathological examination), thereby determining diagnostic sensitivity and specificity of each technique and combinations thereof.

## 2. Materials and Methods

In this retrospective cross-sectional study, the patient collective was composed of all subjects who were operated for their primary OSCC (including uni- or bilateral neck dissection) at the University Hospital for Cranio-Maxillofacial and Oral Surgery of the Medical University of Innsbruck, Austria, between January 2000 and December 2013. For this time period, a preexisting database including detailed clinical and histopathological information was available. In addition to the already available electronic data, all surgical notes providing details with regard to type and extent of the neck dissection (ND) performed were retrieved from the hospital proprietary data system.

To be included in the study sample, patients had to undergo uni- or bilateral ND following preoperative diagnostic imaging by US, CT, MRI, or PET (or any combination thereof).

CT scans with iodinated contrast agent were performed in spiral CT technique with 3 mm coronal and sagittal reconstructions in the soft tissue window and 1 mm reconstructions in the bone window. MRI sequences were as follows: coronal T2 turbo inversion recovery magnitude (TIRM), axial T2 turbo spin echo (tse), axial T1 tse, axial diffusion-weighted imaging (DWI), axial T1 tse Dixon with gadolinium, coronal T1 volume interpolated breathhold examination (VIBE) Dixon with gadolinium.

Patients were excluded as study subjects if treated conservatively only (mainly by radio- and/or chemotherapy).

The primary predictor variable corresponded to the results (score for CT, MRI, and US) of each imaging modality. To radiologically assess the dignity of a lymph node, the score comprising both the cervical lymph node size as well as morphologic criteria was calculated. This assessment was done by one single investigator.

Details of the score were as follows: in accordance with Yoon et al. [13], lymph nodes ≥10 mm were rated positive except for levels I and II where the cut off value was ≥15 mm. Regarding morphology, lymph nodes were rated positive if at least 2 of the following criteria were present: abnormal shape, hilus sign, central necrosis, cluster, peripheral enhancement, architecture and blood flow (US and MRI).

For PET examinations, data were reviewed by a consultant in nuclear medicine (see acknowledgement). The standardized uptake value (SUV) cut off value was 2.

With regard to the primary outcome variable, histologic specimens were considered positive if tumor cells were found in any of the lymph nodes irrespective of size or location. In these cases, the respective side of the neck was rated positive.

All statistical analyses were performed using R statistical package, version R 3.4.2 (R Foundation for Statistical Computing, Vienna, Austria). Continuous variables are expressed as means and standard deviations and categorical variables are stated as numbers and percentages. The diagnostic sensitivity, specificity, positive and negative predictive values, and positive and negative diagnostic likelihood ratios of the different imaging modalities and all possible combinations were calculated relative to the histopathologic results. The 95%-confidence intervals (CI) were calculated as well.

The statistical null hypothesis was that all imaging modalities or combinations would be equal with regard to diagnostic sensitivity and specificity. The alternative hypothesis stated that differences would exist.

All investigations were carried out following the rules of the Declaration of Helsinki of 1975 (available online: https://www.wma.net/what-we-do/medical-ethics/declaration-of-helsinki/, accessed on 27 March 2020), revised in 2013.

This study was independently reviewed and approved by the Ethics Committee of the Medical University of Innsbruck (reference number: AN2014-0184 338/4.11341/8.3).

## 3. Results

From January 2000 to December 2013, a total of 164 patients were diagnosed with OSCC at the University Hospital for Cranio-Maxillofacial and Oral Surgery of the Medical University of Innsbruck, Austria. Ninety-six of these patients underwent uni- or bilateral ND and were therefore included in this study. Seventy-seven patients had an US examination, 95 subjects had a CT scan, 64 an MRI, and 19 a 18F-FDG PET. The number of patients having undergone a combination of two radiological examinations ranged from 15 (MRI+PET) to 76 (US+CT) respective from 13 (US+MRI+PET) to 56 (US+CT+MRI) for three imaging modalities.

The collective of all included 96 patients comprised 37 women (38.5%) and 59 men (61.5%) with a mean age of 59.8 ± 11.7 years (range, 32–87 years) at the time of diagnostic confirmation of the tumor.

During the above-mentioned study period (2000–2013), 22 patients died (22.9%), whereas 74 subjects (77.1%) were still alive at the time of last follow-up.

The localization of the primary tumor is depicted in Table 1. Tumor size and lymph node status according to the TNM classification are shown in Table 2 and Table 3, respectively. Details concerning the histological grading can be found in Table 4.

In 70 cases, ND was performed on the tumor side only (34 on the right and 36 on the left side), whereas 26 patients underwent bilateral ND. Thus, the total number of NDs performed amounts to 122 (60 on the right and 62 on the left side).

The diameter of lymph nodes depicted in US examinations varied between 2.8 mm and 41 mm (mean: 9.21 mm), whereas the respective size ranged from 5 mm to 46 mm in both CT and MRI scans (mean: 13.20 mm and 15.35 mm, respectively).

Regarding sonography, a total of 33 sides were rated positive. Of these, 20 sides (60.6%) were rated positive because of both their size and their morphology, 2 sides (6.1%) were rated positive due to enlarged lymph nodes, and 11 sides (33.3%) were rated positive because of normal-sized but morphologically abnormal lymph nodes.

Regarding computed tomography, a total of 64 sides were rated positive. Of these, 41 sides (64.1%) were rated positive because of both their size and their morphology, 4 sides (6.3%) were rated positive due to enlarged lymph nodes, and 19 sides (29.7%) were rated positive because of normal-sized but morphologically abnormal lymph nodes.

Regarding MRI, a total of 35 sides were rated positive. Of these, 25 sides (71.4%) were rated positive because of both their size and their morphology, 1 side (2.9%) was rated positive due to enlarged lymph nodes, and 9 sides (25.7%) were rated positive because of normal-sized but morphologically abnormal lymph nodes.

Diagnostic sensitivity and specificity as well as the positive and negative predictive values and the positive and negative diagnostic likelihood ratios for all imaging techniques (including all combinations) were calculated and are listed in Table 5.

## 4. Discussion

The aim of this study was to determine how much imaging is minimally needed for valid detection of cervical lymph node metastases in primary OSCC. It was hypothesized that differences regarding sensitivity and specificity of the various imaging modalities (single investigation or combination of techniques) could be revealed. Regarding the sensitivity of 100% alongside with an acceptable specificity, the combinations of CT+MRI or PET+MRI appear to be appropriate for diagnostic imaging in OSCC subjects.

OSCC represents the predominant malignancy in the oral cavity and its incidence has been rising in recent decades [1,2,4]. Men are more likely to be diagnosed with OSSC than women, and the age of diagnosis with this disease is between 45 and 64 years for nearly half of the patients [18,19,20]. As with many other tumor entities, accurate diagnostics including potential involvement of lymph nodes is key for successful management and favorable long-term survival.

One of the most commonly used imaging techniques which still represents a highly valuable diagnostic tool in tumor staging and follow-up is US. The most obvious advantages include widespread availability, favorable tolerance, and absence of radiation exposure. Usually no contrast agent is required. Shortcomings of this technique comprise the limitation to investigate structures deeper than 3–4 cm as well as the lack of reliable differentiation between inflammatory and metastatic lymph nodes. In addition, it is more dependent on investigator skills compared to other modalities like CT or MRI scans.

Well known differences between CT and MRI include the highly accurate depiction of bony structures with the first technique, whereas the latter imaging modality is by far superior regarding the achievable soft tissue contrast. An obvious advantage of MRI examinations is the lack of radiation exposure. Both techniques provide the possibility to evaluate superficial and deep structures (e.g., lymph nodes), although the resolution is limited to approximately 3 mm depending on the protocol used. Standard iodine contrast agents for CT scans bear the risk of renal failure as well as interference with the thyroid gland, whereas the commonly used gadolinium for MRI is usually well tolerated. Allergies to iodine contrast agents are by far more common than adverse reactions to gadolinium or the risk of nephrogenic systemic fibrosis.

In recent decades, there have been great advances with regard to PET examinations. In contrast to the imaging modalities mentioned before, this technique allows for metabolic assessment (with a relatively limited resolution) rather than detailed morphologic depiction of anatomical structures. Therefore, it is nowadays usually combined with CT (or MRI) scans to merge both the anatomical as well as the metabolic diagnostic information with obvious advantages over the use of either technique alone. However, distinguishing between inflammatory and malignant pathologies is not possible in a PET scan. In addition, there is currently no consensus with regard to the achievable sensitivity and specificity of this examination.

In accordance with Lee et al. [21] and Yoon et al. [13], our results showed a higher specificity of US compared to its sensitivity (0.791 vs. 0.543). Regarding the sensitivity of CT scans, our results are higher than data indicated in recent studies which reported a sensitivity between 66% and 86.9% [9,10,13,15,16,17,22]. In contrast, studies describing CT specificity [9,10,13,15,16,22] significantly differ from our results where the specificity (0.639) was lower than the sensitivity (0.950). In other papers—with the exception of publications by Yamazaki et al. and Stoeckli et al. [11,17]—specificity ranged from 74.0% to 99.4% [9,10,13,15] and was thus higher than the respective sensitivity reported.

Results of sensitivity (0.857) and specificity (0.756) of MRI in our analysis are relatively low and comparable to other studies [9,10,13]. Our ratio between sensitivity and specificity is in accordance with results given by Adams et al. and Stuckensen et al. [9,10]. In the publication by Yoon et al. [13], similar results for sensitivity compared to our study were found, however, they indicated a higher specificity.

In contrast to most of the other studies [9,10,12,17], our results showed a higher sensitivity (0.778) compared to specificity (0.625) in PET exams. Only in one study, PET sensitivity was found to be higher than specificity [23].

In our analysis, the highest sensitivity (0.950) was achieved by CT scans, whereas ultrasonography showed the best results for specificity (0.794).

The abundant use of various imaging techniques for staging and follow-up of tumors raises the question of whether different combinations of imaging modalities can demonstrate better diagnostic accuracy than the use of a single technique alone. In the present study, the highest sensitivity was achieved by either a combination of CT and MRI (1.000) or by combining US, CT, and MRI (1.000). In addition, the combination of US and MRI provided the highest specificity (0.643). Unlike Yoon et al. [13], we observed lower values for specificity compared to results for sensitivity.

Combining US, CT, and MRI yielded higher sensitivity compared to CT or MRI alone, however, this combination also entailed a loss of specificity compared to US alone. This result is in contrast to findings by Yoon et al. [13] who reported a higher specificity without loss of sensitivity.

The lack of internationally standardized and accepted criteria for the definition of malignant affection of lymph nodes renders interpretation of radiological findings difficult. Depending on the level, normal lymph nodes can be as large as 10–15 mm. Apart from size, also lymph node architecture, blood flow, and vascularity need to be taken into account. Finally, a conglomerate of lymph nodes has to be interpreted in a different way as compared to a single node. Further challenges arise with regard to variability in the basic concept of evaluation, i.e., patient by patient, ND by ND, or level by level.

Retrospective evaluations always carry the risk of bias, and usually the amount of missing data is more significant than in a prospective study setting. For comparison of radiological results to the gold standard (i.e., histopathological examination of the final ND specimen), only patients who underwent neck dissection could be included. Thus, imaging results of a significant number of patients were not taken into account.

Another limitation of this study is the relatively small sample size which is also reflected by large confidence intervals. For example, although specificity of PET+MRI (70%) seems to be largely superior to the corresponding value of CT+MRI (52%), the respective confidence intervals overlap (i.e., no statistically significant difference detected). This does not necessarily mean that there is no difference but that a potentially existing difference could not be verified, possibly due to a small sample size.

## 5. Conclusions

In view of a 100% sensitivity with a reasonable specificity of 51% and 70% for CT+MRI and PET+MRI, respectively, a standard diagnostic imaging protocol referring to either of these combinations could potentially be sufficient for assessment of cervical lymph nodes in primary OSCC. With regard to its widespread availability, radiologic evaluation using CT and MRI appears to be the most practicable option. 18F-FDG PET/CT may provide better detection of occult lymph node metastasis than CT/MR imaging which potentially improves prognosis and proper management of OCC patients. However, false-positive PET/CT findings resulting in relatively lower specificity compared with that of CT/MR imaging might be a serious disadvantage.

For better comparison of study outcomes in the international literature, official guidelines determining the threshold of size as well as detailed morphological assessment criteria to distinguish normal from malignant lymph nodes are needed.

## Figures and Tables

**Table 1:** Localization of oral squamous cell carcinoma (OSCC).

Localization	Number (*n*)	Percent (%)
floor of the mouth	38	39.6
tongue	13	13.5
cheek	5	5.2
palate	12	12.5
mandible	18	18.8
maxilla	6	6.3
maxillary sinus	1	1.0
lip	2	2.1
oropharynx	1	1.0

**Table 2:** Clinical and pathological classification of tumor size.

T-Stage							
Pathological	pT-Stage	Number (*n*)	Percent (%)	Clinical	cT-Stage	Number (*n*)	Percent (%)
	1	36	37.5		1	16	16.7
	2	38	39.6		2	15	15.6
	3	3	3.1		3	1	1.0
	4	11	11.5		4	8	8.3
	4a	7	7.3		4a	3	3.1
	4b	1	1.0		4b	0	0.0
	N/A	0	0.0		N/A	53	55.2

N/A: data not available.

**Table 3:** Clinical and pathological classification of lymph node status.

*n*-Stage							
Pathological	pN-Stage	Number (*n*)	Percent (%)	Clinical	cN-Stage	Number (*n*)	Percent (%)
	0	60	62.5		0	23	24.0
	1	15	15.6		1	3	3.1
	2	3	3.1		2	3	3.1
	2a	0	0.0		2a	1	1.0
	2b	10	10.4		2b	2	2.1
	2c	8	8.3		2c	1	1.0
	N/A	0	0.0		N/A	63	65.6

N/A: data not available.

**Table 4:** Grading.

Grading	Number (*n*)	Percent (%)
1	10	10.4
2	62	64.6
3	22	22.9
*n*/A	2	2.1

*n*/A: data not available.

**Table 5:** Comprehensive overview including all imaging modalities. Key results relevant for our conclusions are highlighted in bold.

	Sensitivity	Specificity	PPV	NPV	PDLR	NDLR
US	0.543	**0.794**	0.576	0.771	2.637	0.576
	(95% CI	**(95% CI**	(95% CI	(95% CI	(95% CI	(95% CI
	[0.378–0.708])	**[0.698–0.890])**	[0.407–0.744])	[0.673–0.870])	[1.510–4.603])	[0.393–0.842])
CT	0.950	0.639	0.594	0.958	2.631	0.078
	(95% CI	(95% CI	(95% CI	(95% CI	(95% CI	(95% CI
	[0.882–1.000])	[0.528–0.750])	[0.473–0.714])	[0.902–1.000])	[1.919–3.606])	[0.020–0.305])
MRI	0.857	0.756	0.686	0.895	3.506	0.189
	(95% CI	(95% CI	(95% CI	(95% CI	(95% CI	(95% CI
	[0.727–0.987])	[0.630–0.881])	[0.532–0.840])	[0.797–0.992])	[2.053–5.990])	[0.075–0.476])
PET	0.778	0.625	0.538	0.833	2.074	0.356
	(95% CI	(95% CI	(95% CI	(95% CI	(95% CI	(95% CI
	[0.506–1.000])	[0.388–0.862])	[0.267–0.809])	[0.622–1.000])	[1.007–4.273])	[0.099–1.279])
US+CT	0.970	0.476	0.492	0.968	1.851	0.064
	(95% CI	(95% CI	(95% CI	(95% CI	(95% CI	(95% CI
	[0.911–1.000])	[0.353–0.560])	[0.371–0.614])	[0.906–1.000])	[1.452–2.361])	[0.009–0.446])
US+MRI	0.875	0.643	0.583	0.900	2.450	0.194
	(95% CI	(95% CI	(95% CI	(95% CI	(95% CI	(95% CI
	[0.743–1.000])	[0.498–0.788])	[0.422–0.744])	[0.792–1.000])	[1.589–3.778])	[0.066–0.574])
US+PET	0.857	0.533	0.462	0.889	1.837	0.268
	(95% CI	(95% CI	(95% CI	(95% CI	(95% CI	(95% CI
	[0.598–1.000])	[0.281–0.786])	[0.191–0.733])	[0.684–1.000])	[0.988–3.414])	[0.041–1.747])
CT+MRI	**1.000**	**0.512**	0.556	1.000	2.050	0.000
	**(95% CI**	**(95% CI**	(95% CI	(95% CI	(95% CI	(95% CI
	**[1.000–1.000])**	**[0.359–0.665])**	[0.410–0.701])	[1.000–1.000])	[1.498–2.805])	[0.000–0.000])
CT+PET	0.875	0.461	0.500	0.857	1.625	0.271
	(95% CI	(95% CI	(95% CI	(95% CI	(95% CI	(95% CI
	[0.646–1.000])	[0.190–0.732])	[0.238–0.762])	[0.598–1.000])	[0.921–2.866])	[0.039–1.857])
MRI+PET	**1.000**	**0.700**	0.700	1.000	3.333	0.000
	**(95% CI**	**(95% CI**	(95% CI	(95% CI	(95% CI	(95% CI
	**[1.000–1.000])**	**[0.416–0.984])**	[0.416–0.984])	[1.000–1.000])	[1.293–8.591])	[0.000–0.000])
US+CT+MRI	**1.000**	0.394	0.489	1.000	1.652	0.000
	**(95% CI**	(95% CI	(95% CI	(95% CI	(95% CI	(95% CI
	**[1.000–1.000])**	[0.239–0.550])	[0.343–0.635])	[1.000–1.000])	[1.278–2.136])	[0.000–0.000])
US+CT+PET	0.857	0.385	0.429	0.833	1.393	0.371
	(95% CI	(95% CI	(95% CI	(95% CI	(95% CI	(95% CI
	[0.598–1.000])	[0.120–0.649])	[0.169–0.688])	[0.535–1.000])	[0.823–2.356])	[0.053–2.586])
US+MRI+PET	**1.000**	0.600	0.555	1.000	2.500	0.000
	**(95% CI**	(95% CI	(95% CI	(95% CI	(95% CI	(95% CI
	**[1.000–1.000])**	[0.296–0.904])	[0.231–0.880])	[1.000–1.000])	[1.170–5.341])	[0.000–0.000])
CT+MRI+PET	**1.000**	0.500	0.600	1.000	2.000	0.000
	**(95% CI**	(95% CI	(95% CI	(95% CI	(95% CI	(95% CI
	**[1.000–1.000])**	[0.153–0.846])	[0.296–0.904])	[1.000–1.000])	[1.000–3.999])	[0.000–0.000])
US+CT+MRI+PET	**1.000**	0.375	0.500	1.000	1.600	0.000
	**(95% CI**	(95% CI	(95% CI	(95% CI	(95% CI	(95% CI
	**[1.000–1.000])**	[0.039–0.710])	[0.190–0.810])	[1.000–1.000])	[0.935–2.737])	[0.000–0.000])

PPV: positive predictive value; NPV: negative predictive value; PDLR: positive diagnostic likelihood ratio; NDLR: negative diagnostic likelihood ratio.
